# Copper toxicity leads to accumulation of free amino acids and polyphenols in *Phaeodactylum tricornutum* diatoms

**DOI:** 10.1007/s11356-023-25939-0

**Published:** 2023-02-21

**Authors:** Paula Santiago-Díaz, Argimiro Rivero, Milagros Rico, Aridane González González, Melchor González-Dávila, Magdalena Santana-Casiano

**Affiliations:** 1 Departamento de Química, Facultad de Ciencias del Mar, Universidad de Las Palmas de Gran Canaria, Campus de Tafira, 35017 Las Palmas de Gran Canaria, Spain; 2 Instituto de Oceanografía Y Cambio Global (IOCAG), Universidad de Las Palmas de Gran Canaria, Las Palmas de Gran Canaria, Spain

**Keywords:** Amino acid, Polyphenol, Microalgae, Antioxidant activity, Copper toxicity, *Phaeodactylum tricornutum*

## Abstract

This work is focused on the effect of lethal and sub-lethal copper (Cu) concentrations on the free amino acid and polyphenol production by the marine diatom *Phaeodactylum tricornutum* (*P. tricornutum*) after 12, 18, and 21 days of exposure. The concentrations of 10 amino acids (arginine, aspartic acid, glutamic acid, histidine, lysine, methionine, proline, valine, isoleucine, and phenylalanine) and 10 polyphenols (gallic acid, protocatechuic acid, *p*-coumaric acid, ferulic acid, catechin, vanillic acid, epicatechin syringic acid, rutin, and gentisic acid) were measured by RP-HPLC. Under lethal doses of Cu, free amino acids reached levels significantly higher than those in the control cells (up to 21.9 times higher), where histidine and methionine showed the highest increases (up to 37.4 and 65.8 times higher, respectively). The total phenolic content also increased up to 11.3 and 5.59 times higher compared to the reference cells, showing gallic acid the highest increase (45.8 times greater). The antioxidant activities of cells exposed to Cu were also enhanced with increasing doses of Cu(II). They were evaluated by 2,2-diphenyl-1-picrylhydrazyl (DPPH) free radical scavenging ability (RSA), cupric ion reducing antioxidant capacity (CUPRAC), and ferric reducing antioxidant power (FRAP) assays. Malonaldehyde (MDA) exhibited the same tendency: cells grown at the highest lethal Cu concentration yielded the highest MDA level. These findings reflect the involvement of amino acids and polyphenols in protective mechanisms to overcome the toxicity of copper in marine microalgae.

**Graphical Abstract:**

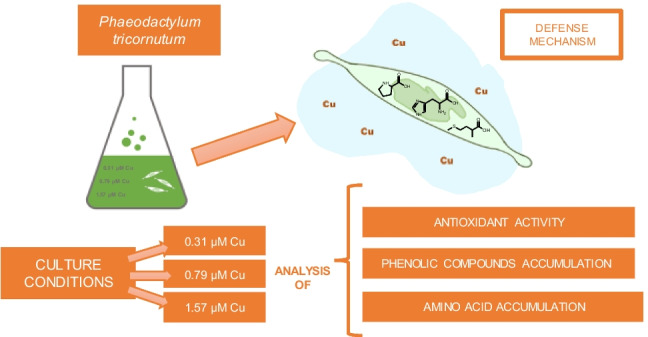

**Supplementary Information:**

The online version contains supplementary material available at 10.1007/s11356-023-25939-0.

## Introduction

The presence of heavy metals in the coastal seawater at high concentrations has a direct impact on marine microorganisms. The uptake of metals by the cells can cause a range of toxic alterations such as enhanced production of reactive oxygen species (ROS) inducing oxidative stress and breaking the oxidative balance of microalgae; disruption of protein structures affecting enzymes and nucleic acids functions by metal binding or displacement of an essential element, among others (Shivaji and Dronamaraju 2019; Wei et al. 2014). Coastal waters are critical habitats for many marine species, where metal toxicity can change the start and end timing of the blooms and their amplitude, affecting the survival and hatching time of commercially important species (Trombetta et al. 2019).

Copper (Cu) is one of those trace metals that becomes toxic when exceeding certain levels. Natural concentrations of Cu in coastal seawater are generally low (0.008 and 0.050 μM); however, local anthropogenic sources principally related with industrial activities and domestic wastes can result in local increase in Cu concentrations above 3.0 μM (Leal et al. 2018; Pérez-Cid et al. 2021). According to the aquatic life criteria from Environmental Protection Agency (EPA), the Criterion Maximum Concentration (CMC) for Cu acute toxicology is 0.075 μM and the Criterion Continuous Concentration (CCC) for Cu chronic toxicology is 0.049 μM.

There is considerable evidence with respect to the involvement of phenolic compounds in protection mechanisms against copper toxicity in plants and algae growing in conditions of metal stress (Kováčik et al. 2010; López et al. 2015; Lwalaba et al. 2020; Sharma et al. 2019; Stiller et al. 2021). However, little is known about the implication of amino acids. Therefore, this study is mainly focused on the impact of copper toxicity on the amino acid profile of microalgae cells during growth. Some studies have evidenced accumulation of free amino acids in microalgae cells cultivated under Cu concentrations as a response to heavy metal stress (Hamed et al. 2017; Seregin and Kozhevnikova 2021).

Amino acids like histidine (His) and methionine (Met) have been found to play a significant role in heavy metal detoxification and tolerance (Hall 2002; Murphy et al. 2011; Patrick 2003). His, Met, and cysteine (Cys) are the predominant amino acids in several transmembrane proteins to facilitate Cu transport across the cell membrane at different pH and oxidative environments (Rubino et al. 2011; Rubino and Franz 2012; Öhrvik and Thiele 2014). After cell wall metal adsorption, the transport toward chloroplasts, mitochondria, and vacuoles is mediated by small glutamic acid (Glu) and Cys-rich polypeptides, namely, metallothioneins and phytochelatins (Balzano et al. 2020; Blaby-Haas and Merchant 2012; Cobbett 2000). These polypeptides have been evidenced in *P. tricornutum* growing under exposure to metals (Morelli et al. 2005; Torres et al. 1997; Wei et al. 2014).

Microalgae can provide information on the potential impacts of Cu. The marine diatom *P. tricornutum* is commonly used in toxicity tests (Moreno-Garrido et al. 2000; Singh and Shrivastava 2016). Its sensitivity makes them ideal to study the influence of Cu levels on the phenolic and amino acid profiles variations at different phases of the diatom growth. However, the biochemical response of these microorganisms cultivated under Cu stress is still not fully understood, neither their role in regulating the speciation of trace metals contaminants in seawater as components of the dissolved organic matter.

The aim of this work was to study the chemical response of the diatom *P. tricornutum* to high Cu concentrations at different stages of growth (12, 18, and 21 days) by analyzing the contents of 10 amino acids and 10 polyphenols in cells. The changes in composition could be used by cells as an intracellular mechanism of reaction to the Cu toxicity. Moreover, the antioxidant activities of *P. tricornutum* cells extracts were evaluated in terms of radical scavenging ability (RSA), the total reduction power by the ferric reducing antioxidant power (FRAP) assay, and the cupric ion reducing antioxidant capacity with (CUPRAC) assay. In addition, malonaldehyde (MDA) was also measured as a biomarker for oxidative stress (Hodges et al. 1999).

## Materials and methods

### Chemicals

Methanol (HPLC gradient grade), ethanol, and tetrachloroethylene (synthesis grade) were purchased from Scharlab (Barcelona, Spain). CuSO_4_·7H_2_O, phenylisothiocyanate (PITC), 2,4,6-tri(2-pyridyl)-triazine (TPTZ), neocuproine, and DPPH were supplied by Sigma-Aldrich (St. Louis, MO, USA). Triethylamine (TEA), hydrochloric acid (37%), sodium acetate, glacial acetic acid, Fe_3_Cl·6H_2_O and FeSO_4_·7H_2_O, trichloroacetic acid (TCA), butylated hydroxytoluene (BHT), and 2-thiobarbituric acid (TBA) (analysis quality) were supplied by Panreac (Barcelona, Spain). Formic acid (synthesis grade) and amino acid standards Glu, His, Met, arginine (Arg), aspartic acid (Asp), lysine (Lys), proline (Pro), valine (Val), isoleucine (Ile), and phenylalanine (Phe) were provided by Merck (Darmstadt, Germany). Polyphenol standards were supplied as follows: gallic acid (GAL), protocatechuic acid (PCA), p-coumaric acid (COU), ferulic acid (FA), catechin (CAT), vanillic acid (VAN), epicatechin (ECAT), syringic acid (SYR), and Trolox (TR) by Sigma-Aldrich Chemie (Steinheim, Germany); rutin (RU) and gentisic acid (GA) by Merck (Darmstadt, Germany). Ultrapure water was obtained from a Milli-Q system from Millipore (Bedford, MA, USA).

### Algae cultivation

Axenic strains of *P. tricornutum* (REC 001B) were provided by the Spanish Bank of Algae (Taliarte, Spain). The diatoms were harvested in a clean culture chamber (Friocell FC111) with permanent illumination (24 h at 8000 lx) under a constant temperature of 24 °C.

Seawater used for cultures was sampled off the coast of Gran Canaria, treated with ultraviolet radiation and passed through 0.45-µm filters. The seawater was enriched with nutrients (SWn) at the concentrations used in a f/2 medium without added trace metals and EDTA. The nutrient concentrations were [NO_3_^−^] = 883 μM, [HPO_4_^2−^] = 29.3 μM, and [SiO_3_^2−^] = 142 μM (González et al. 2012).

### Cu experiments

For the Cu exposure treatments, cultures of SWn with an initial cell density of 1 × 10^7^ cells L^−1^ were spiked with 0.31 µM, 0.79 µM, and 1.57 µM Cu (II). The Cu concentrations selected were lower than those described in coastal seawater (Leal et al. 2018) but higher than the CMC and CCC defined by EPA for acute and chronic toxicology, respectively.

The control was a SWn culture with the same initial cell density without Cu addition. To separate the biomass, the cells were filtered by gravity to avoid rupture using 1.2-µm filters (trace metal acid clean pore-size nitrocellulose, Sartorius™). This process was carried out after 12, 18, and 21 days of growth. The cell concentration was determined daily with a light microscope (Microbiotest, Inc.) with a hemocytometer counter and by measuring the absorbance (Abs) at 670 nm with a spectrophotometer (USB4000).

### Free amino acid extraction and quantification

The following 10 amino acids were examined in the cells: Glu, His, Met, Arg, Asp, Lys, Pro, Val, Ile, and Phe. They were selected because of their antioxidant properties (Hwang et al. 2019) and their role in the response of plants to several types of abiotic stress (Marquis et al. 2020; Zhang et al. 2020a, b).

Samples (1 L) of control and Cu-exposed diatom cultures were filtered as described above, and the biomass was extracted with deionized water (50 mL) by sonication for 30 min. The mixture was freeze-dried, and the residue was dissolved in 5 mL of water. Amino acids were derivatized according to Shi et al. (2013). In brief, samples or amino acid standard solutions (5 mL) were mixed with 2.5 mL of PITC 0.1 M and 2.5 mL of TEA 1 M, and the resulting solutions were stirred for 1 h at room temperature. Then, 5 mL of tetrachloroethylene was added and vigorously shaken, and the upper layer was collected. This step was performed twice. The final solution was filtered through a 0.22-µm nylon and stored at − 20 °C until analysis.

Chromatographic analysis was carried out with a Jasco LC-4000 HPLC equipment provided with a PU-4180 quaternary pump, an AS-4150 autosampler, an MD-4015 photodiode array detector and an LC-Net ll interface. Amino acids were separated with a Phenomenex C18 column (250 mm × 4.6 mm, 5 µm) and a Phenomenex guard column maintained at 30 °C. The gradient elution was made with ultrapure water with 0.1% formic acid as mobile phase A and methanol as mobile phase B. The flow rate was 1 mL min^−1^ and the injection volume was 10 µL. The following program for eluent A was applied: 0 min, 75%; 30 min, 40%; 40 min, 40%; and finally, column was washed and reconditioned (Santiago-Díaz et al. 2022). Data acquisition was carried out with ChromNav software, and the statistical analysis (available as supplementary data) was made using Past software provided with an ANOVA test and a Tukey’s test. Significant differences between Cu treatments for each amino acid after 12 and 18 days were found. The determination of statistically significant differences (considered at *p* < 0.05) between each treatment and the control for each individual amino acid was carried out with the Tukey’s test. Algae samples were analyzed by triplicate and the results were expressed as fmol cell^−1^.

### Polyphenols contents, antioxidant activities, and MDA determinations

Samples (1 L) exposed to Cu for 18 days were filtered as described above and cells were freeze-dried and extracted twice with 10 mL of methanol combining sonication and stirring. The samples were centrifuged at 2700 g for 15 min, the supernatants were collected and evaporated to dryness, and the residue was dissolved in 5 mL of methanol for determining polyphenols contents and antioxidant activities. For MDA analysis, freeze-dried cells were extracted twice with deionized water (3 mL).

The concentrations of the 10 polyphenols GAL, PCA, CAT, VAN, RU, ECAT, SYR, GA, CA, and FA were evaluated. They were selected because their involvement in protection mechanisms against copper toxicity has been widely evidenced (Kováčik et al. 2010; López et al. 2015; Lwalaba et al. 2020; Rico et al. 2013; Santana-Casiano et al. 2014). Methanol extracts described above (2 mL) were concentrated to dryness and the residue was dissolved in methanol (200 μL) and filtered with a syringe filter (0.2 µm). The chromatographic analysis was made with the equipment and columns described in the “Free amino acid extraction and quantification” section. The elution was also performed with the same eluents, flow rate, injection volume, and column temperature (30 °C). The gradient elution method for A was from 0 to 5 min, 80% isocratic; from 5 to 30 min, linear gradient from 80 to 40%; and the column was washed with a mixture of A and B (1:9) and conditioned for the next analysis. Simultaneous monitoring was set at 270 nm (GAL, PA, CAT, VA, RU, ECAT, and SA) and 324 nm (GA, CA, and FA) for quantification (Santiago-Díaz et al. 2022). Algae samples were analyzed by triplicate, and the results were expressed as attomol (amol) cell^−1^.

The RSA was evaluated according to Bondet et al. (1997) with modifications. Methanol extracts (70 µL) were mixed with 1 mL of free radical DPPH solution (0.067 mM) for 10 min. The Abs was recorded at 515 nm, and the results obtained from a calibration curve (*y* = 11.987x—0.1352, *R*^2^ = 0.9996) were expressed as fmol of inhibited DPPH cell^−1^. Measurements were taken in triplicate and the results were averaged.

The FRAP reagent was freshly prepared by mixing 100 mL of acetate buffer solution 0.3 M (pH 3.6) with 10 mL of TPTZ (10 mM) in HCl (40 mM) and 10 mL of FeCl_3_·6H_2_O solution (20 mM) (Benzie and Strain 1996). Methanol extracts (200 μL) and 1.4 mL of pre-warmed FRAP reagent (37 °C) were mixed for 10 min. The mixture was cooled and the Abs recorded at 593 nm. Results calculated from a calibration curve constructed with FeSO_4_·7H_2_O solutions ranging from 0.38 to 2.69 µM (*y* = 0.6265x + 0.1882, *R*^*2*^ = 0.9988) were expressed as pmol of Fe(II) cell^−1^.

The CUPRAC reagent was freshly prepared with equal volumes of CuSO_4_·7H_2_O (10 mM), neocuproine ethanolic solution (7.5 mM), and NH_4_Ac buffer solution (1 M) (Apak et al. 2006). This reagent (570 µL) was diluted with water (930 µL) and mixed with 200 μL of methanol extracts for 30 min, and the Abs was recorded at 450 nm. The results were expressed as fmol TR cell^−1^ and calculated from a standard curve prepared with solutions in the range of concentrations from 11.6 to 77.5 µM (*y* = 0.6215x + 0.0004; *R*^2^ = 0.9996). The estimation was carried out in triplicate, and the results were averaged.

Aqueous extracts of cells (0.5 mL) were mixed with either (i) 0.5 mL of aqueous solution of TCA (20%) and BHT (0.01%) or (ii) 0.5 mL of the above solution with 0.5% TBA added. The mixtures were heated at 95 °C for 25 min, cooled to 5 °C, and centrifuged at 1300 rpm for 10 min. MDA reacts with TBA to produce a pinkish-red adduct. The Abs was measured at three wavelengths to correct the interferences of carbohydrates and pigments (440, 532, and 600 nm) and the equivalents of MDA were calculated according to Hodges et al. (1999), and expressed as amol of MDA cell^−1^.

## Results

### Effects of Cu levels on growth of P. tricornutum

**Fig. 1:**
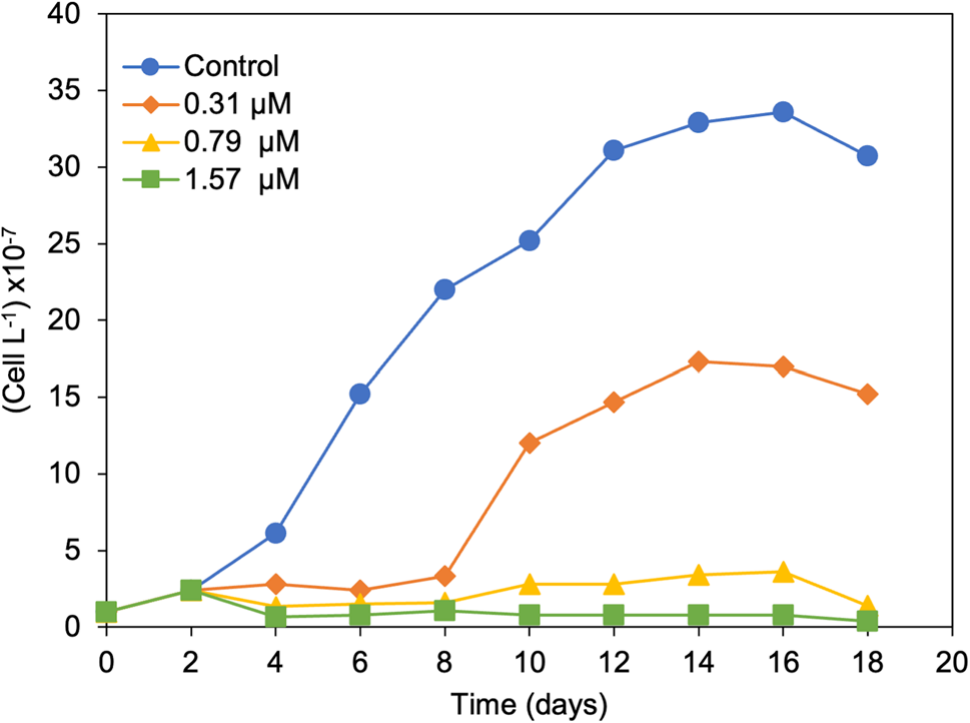
Growth curve of *P. tricornutum* exposed to different copper concentrations in seawater enriched with f/2 nutrients (24 h at 8000 lx, 24 °C) with an initial cell density of 1 × 10^7^ cells L.^−1^

The growth curves of *P. tricornutum* in controls and exposures of 0.31, 0.79, and 1.57 µM of Cu are shown in Fig. 1. In the controls, the mean absolute growth rate was 2.11 × 10^7^ cell L^−1^ day^−1^. The stationary phase ended after 16 days, achieving during this phase a maximum cell density of 33.6 × 10^7^ cell L^−1^. From 16 to 21 days, the diatoms cells were in the death phase. Therefore, the cell density was daily measured until day 18 and subsequently measured on day 21. According to the evolution of the control cultures, the days 12, 18, and 21 were selected for the determination of free amino acid profiles and the day 18 for the studies of antioxidant activity, MDA, and phenolic contents.

When the initial concentration of Cu was 0.31 µM, a prolonged lag phase was found compared to the control culture. Although the mean absolute growth rate in 0.31 µM Cu cultures (0.783 × 10^7^ cell L^−1^ day^−1^) was significantly different (*ρ* < 0.05) from the control culture, biomass was reduced only by 37%, suggesting that this concentration is a sub-lethal dose to the microalga. However, for cultures enriched with 0.79 and 1.57 µM of Cu, lack of exponential phase of growth was observed and cell density decreased by 82 and 91% respectively, indicating these concentrations are lethal for *P. tricornutum* under the experimental conditions.

### Free amino acid profile in P. tricornutum

The amino acid profiles of *P. tricornutum* cells collected after 12, 18 and 21 days are summarized in Table 1. Under lethal Cu concentrations, the total sum of free amino acid contents quantified in diatom cells increased with the addition of Cu to the culture medium, reaching levels up to 21.9 times higher compared to the control culture. In these conditions, Met and His were the predominant amino acids, showing the greatest increase in concentration up to 66.8 and 24.5 times higher respectively. Increased concentrations of Glu (up to 20.5 times higher) and Pro (up to 22 times higher) were also observed.

**Table 1:** Free amino acid contents in extracts of the diatom *P. tricornutum* exposed to different Cu concentrations after 12, 18, and 21 days

Amino acid^a^	Control	[Cu(II)] 0.31 μM	[Cu(II)] 0.79 μM	[Cu(II)] 1.57 μM
After 12 days				
His	4.64 ± 0.54	9.04 ± 0.12^b^	28.3 ± 0.8^b^	57.5 ± 0.7^b^
Arg	0.41 ± 0.01	1.05 ± 0.00^b^	1.45 ± 0.33^b^	n.d
Glu	0.54 ± 0.01	0.96 ± 0.00^b^	2.16 ± 0.01^b^	4.43 ± 0.05^b^
Asp	0.58 ± 0.00	1.18 ± 0.01^b^	2.94 ± 0.02^b^	7.54 ± 0.04^b^
Pro	0.38 ± 0.00	0.83 ± 0.03^b^	1.71 ± 0.27^b^	3.08 ± 0.06^b^
Met	2.08 ± 0.02	8.33 ± 0.69	84.9 ± 1.7^b^	136.9 ± 8.6^b^
Val	0.56 ± 0.00	1.28 ± 0.01^b^	n.d	n.d
Lys	0.92 ± 0.04	1.04 ± 0.03^b^	n.d	n.d
Sum	10.11 ± 0.62	23.71 ± 0.89	121.40 ± 3.13	209.40 ± 9.45
After 18 days				
His	2.31 ± 0.09	6.21 ± 0.06^b^	56.5 ± 0.3^b^	86.4 ± 2.0^b^
Arg	1.96 ± 0.06	4.14 ± 0.10^b^	20.6 ± 0.2^b^	n.d
Glu	0.42 ± 0.02	0.87 ± 0.01^b^	4.94 ± 0.08^b^	8.62 ± 0.03^b^
Asp	n.d	0.52 ± 0.01	12.4 ± 0.1	11.7 ± 0.1
Pro	0.38 ± 0.02	0.63 ± 0.01	3.31 ± 0.10	8.41 ± 0.04^b^
Met	1.22 ± 0.01	3.25 ± 0.13	69.2 ± 4.7^b^	29.9 ± 3.9^b^
Val	1.16 ± 0.02	0.33 ± 0.00^b^	n.d	n.d
Lys	0.38 ± 0.02	0.65 ± 0.03^b^	4.85 ± 0.17^b^	n.d
Sum	7.83 ± 0.24	16.6 ± 0.3	171.8 ± 5.7	145.0 ± 6.1
After 21 days				
His	11.8 ± 0.0	24.7 ± 0.1^b^	37.4 ± 0.4^b^	45.0 ± 0.2^b^
Arg	1.14 ± 0.04	1.97 ± 0.04^b^	2.82 ± 0.04^b^	2.22 ± 0.18^b^
Glu	1.75 ± 0.03	2.26 ± 0.01^b^	3.11 ± 0.08^b^	3.29 ± 0.02^b^
Asp	1.84 ± 0.02	2.53 ± 0.03^b^	3.90 ± 0.04^b^	4.70 ± 0.02^b^
Pro	1.12 ± 0.05	1.83 ± 0.09^b^	2.62 ± 0.14^b^	2.26 ± 0.05^b^
Met	8.32 ± 0.11	16.9 ± 0.93^b^	22.5 ± 1.5^b^	84.5 ± 5.7^b^
Val	1.61 ± 0.01	2.66 ± 0.07^b^	3.86 ± 0.05^b^	3.55 ± 0.05^b^
Lys	1.43 ± 0.00	2.00 ± 0.00^b^	2.82 ± 0.03^b^	n.d
Sum	29.0 ± 0.3	54.9 ± 1.3	79.0 ± 2.3	145.6 ± 6.2

n.d. means not detected

^a^Fmol of amino acid per cell (means ± standard deviation of three measurements)

^b^Significant differences to the control (Tukey’s test, *p* < 0.05)

Among the analyzed free amino acids, His, Glu, Pro, and Met were detected in all the extracts. However, Ile and Phe were below detection limit under the experimental conditions (not presented in the Table 1). Val was only detected after 12 and 18 days of exposure to sub-lethal Cu concentrations, and detected in all the cultures after 21 days; Lys was not detected in cells harvested after 12 days at 0.79 µM of Cu, and after 18 and 21 days of exposure to the highest Cu concentration (1.57 μM).

### Phenolic profile in P. tricornutum cells

Profile of polyphenols, measured as content of ten polyphenols in cell extracts of *P. tricornutum* biomass exposed to copper for 18 days, was strongly affected by the metal concentration (Table 2). Under our experimental conditions, ECAT was not detected in the seawater enriched with 0.79 µM of Cu and CAT was only detected in the control cells. GA, VAN, and SYR were detected in all cultures. The other 5 polyphenols investigated were below detection limit. As compared to the control, the amounts of individual phenolic compounds GA, VAN, and SYR increased strongly at the lethal Cu concentration of 1.57 µM. The same behavior was observed in the total sum of the identified polyphenols, which was 11.3 times higher.

**Table 2:** Polyphenol contents in cells of diatom *P. tricornutum* exposed to different copper levels for 18 days (expressed as amol cell^−1^)

Phenolic compound*	Control	Cu(II) 0.31 µM	Cu(II) 0.79 µM	Cu(II) 1.57 µM
Gallic acid	10.2 ± 4.0	122.8 ± 34.0	283.3 ± 16.5	467.0 ± 31.5
Catechin	11.8 ± 4.1	n.d	n.d	n.d
Vanillic acid	24.0 ± 1.6	14.2 ± 2.5	53.3 ± 3.8	132.0 ± 5.6
Syringic acid	10.0 ± 1.9	8.27 ± 1.99	20.6 ± 3.1	23.7 ± 0.4
Epicatechin	7.59 ± 3.95	3.13 ± 0.32	n.d	100.6 ± 17.4
Sum	63.9 ± 15.5	148.4 ± 38.7	357.2 ± 23.4	723.2 ± 55.0

n.d. means not detected

*Results are expressed as amol cell^−1^ (means ± standard deviation of three measurements)

### Antioxidant activities

The antioxidant activities of cells exposed to Cu for 18 days and evaluated by DPPH scavenging activity, FRAP and CUPRAC tests were enhanced as the concentration of Cu increased (Table 3)*.* The reference cells yielded enough antioxidants to inhibit 1.4 fmol of DPPH cell^−1^ followed by cells cultured at 0.31, 0.79, and 1.57 µM of Cu. FRAP and CUPRAC results showed the same tendency: cells grown at the highest lethal Cu level yielded the highest antioxidant capacities.

**Table 3:** Antioxidant activities and MDA contents per cell of *P. tricornutum* cultured under copper exposition for 18 days

Cu concentration	FRAP*	CUPRAC*	Inhibited DPPH*		MDA*
(µM)	(fmol of Fe(II))	(pmol of TR)	(fmol)		(amol)
0	10.44 ± 0.02	6.38 ± 0.02	1.4 ± 0.2	4.5 ± 0.4	
0.31	11.08 ± 0.01	10.55 ± 0.02	26.5 ± 0.5	10.25 ± 0.07	
0.79	29.06 ± 0.07	29.53 ± 0.05	59 ± 2	23.7 ± 0.1	
1.57	31.1 ± 0.2	41.8 ± 0.2	79 ± 1	65.8 ± 0	

*All results are expressed per cell as means ± standard deviation of three measurements

### MDA contents

The amount of cellular MDA increased up to 14.5-fold higher than that of the control at the highest lethal level of Cu, reflecting increased oxidative stress (Table 3).

## Discussion

### Effects of Cu levels on growth of P. tricornutum

The studies carried out showed that the increase in Cu concentration produced a delay in the lag phase and a lower absolute growth rate in the exponential phase that resulted in a lower number of cells in the stationary phase under sub-lethal Cu doses (Fig. 1). The average cell number was close to the starting cell concentration when the Cu levels exceeded the toxicity threshold (0.79 uM and 1.57 uM), indicating an inhibitory effect, as has been previously reported (López et al. 2015; Osborn and Hook 2013; Renzi et al. 2014; Rico et al. 2013). These results agree with the study of Markina and Aizdaicher (2006), who found a decrease in *P. tricornutum* cell concentration grown on Goldberg medium prepared from seawater and an extended lag phase with increasing levels of Cu (2.04 and 3.93 µM of Cu). This behavior was also reported by Cid et al. (1995), and only Cu concentrations higher than 15.74 µM did not fit the logistic function.

The initial cell density used in this study has been reached in some coastal areas (Dursun and Tas 2019) and in lake ecosystems (Zhang et al. 2020a, b). Lower phytoplankton abundances commonly observed in coastal waters (Effendi et al. 2016; Fehling et al. 2012) could intensify the toxic effects of Cu exposition (Moreno-Garrido et al. 2000; Singh and Shrivastava 2016) affecting the physiological processes of coastal marine organisms and their communities (Leal et al. 2018; Trombetta et al. 2019).

### Free amino acids profile of cells

The evolution of each single free amino acid content in the control cells during growth is shown in Table 1. The maximum cell density was achieved on the stationary phase, where cells showed a lower total content of amino acids (7.83 fmol cell^−1^) than that found at the end of the exponential phase (10.11 fmol cell^−1^). Several studies do agree with this temporal variability of free amino acids observed here, indicating that this reduction of amino acids is due to the formation of proteins involved in all cell functions when the maximum biomass is reached (Lourenço et al. 2004; Vendruscolo et al. 2019). Diatoms *Rhizosolenia delicatula* have shown the same behavior during a spring bloom, where the total free amino acids decreased when the maximum biomass was achieved (Martin-Jézéquel et al. 1992). These same results were also observed by Sakevich and Klochenko (1998), who studied the variability of the free amino acids profile of cyanobacteria *Microcystis aeruginosa* and green microalgae *Scenedesmus acuminatus* during growth, and reported higher amino acid contents at the early growth exponential phase, which decreased at the stationary phase. In the present study, the free amino acid concentrations increased after 21 culture days probably due to the degradation of proteins in dead cells (Bidle 2016).

Free amino acids in cells grown in seawater enriched with a sub-lethal dose of Cu exhibited similar behavior than the control cells. Reduction of free amino acids was observed during the highest biomass accumulation growth phase, followed by an increase in the death phase after 21 days (Table 1), which could be due to proteolysis according to Sakevich and Klochenko (1998) and Martin-Jézéquel et al. (1992). However, the free amino acid levels at sub-lethal Cu dose were much higher than those found in the control cells (Table 1). We hypothesize that this accumulation of free amino acids could be a self-defense mechanism to minimize Cu toxicity as has been previously reported (Djoko et al. 2017; Kovács et al. 2012; Lwalaba et al. 2020).

In contrast to the free amino acids behavior described above for control cells, a dramatic increase of free amino acids was observed in cells exposed to both lethal Cu levels (Table 1). These results agree with the findings of Jaishankar and Srivastava (2017), who reported that the entry of microorganisms to the stationary phase survival caused by the presence of toxicants or other stress factors ceases growth but cells remain metabolically active. These authors indicated that cells activate the mechanisms to adapt and survive at this stage, reprograming the gene expression pattern and diverting their resources to increase the amino acids production rather than cell division until environmental conditions improve, with an overall decrease in protein synthesis. This reduction of protein synthesis has been reported as a common consequence of stress, remaining the amino acids free and increasing their concentration in the intracellular medium (Kovács et al. 2012; Lwalaba et al. 2020; Sui et al. 2019). Accumulation of free amino acids was also reported by Afkar et al. (2010), who evaluated the effect of cobalt, copper, and zinc on the physiological response of green microalgae *Chlorella vulgaris*, where cells exposed to Cu below a concentration of 0.1 µM showed the most pronounced stimulation of the total free amino acid production.

Among the tested free amino acids here, the concentration of Met and His experienced the major increase at the lethal Cu doses (Table 1). These increases could be linked to their ability of binding metal ions to transport them across the cell membrane and to prevent metals entry into the cell (Narayanan and Natarajan 2018; Öhrvik and Thiele 2014). Combinations of Met/His or Met/Cys can facilitate Cu regulation under different scenarios of pH and redox environments (Rubino et al. 2011; Rubino and Franz 2012). Kováčik et al. (2010) also reported accumulation of amino acids Met, His, Arg, and Pro in microalgae *Scenedesmus quadricauda* exposed to 25 μM of Cu.

Cells exposed to Cu also showed higher concentrations of free Glu and Pro than those found in the control cells (Table 1). These increased levels of free Pro (osmolyte, free radical scavenger, and metal chelator) and its precursor Glu can also contribute to mitigate Cu stress (Çelekli et al. 2013; Tripathi and Gaur 2004). Pro detoxifies the ROS excess produced under stress, and its accumulation has been considered an important index for stress tolerance capacity in plants, bacteria, algae, and other organisms (Hamed et al. 2017; Sharma and Dietz 2006). Accumulation of intracellular Pro has also been detected in microalgae *Chlorella sorokiniana* and *Scenedesmus acuminatus* exposed to sub-lethal doses of Cu (Hamed et al. 2017), in cyanobacteria *Westiellopsis prolifica* under several heavy metal stresses (Fatma et al. 2007), and in plants treated with Cu and Co, among others (Lwalaba et al. 2020).

### Polyphenols profile of cells, antioxidant activities, and MDA content

After 18 days of exposition to lethal Cu concentrations, the contents of all identified polyphenols increased (Table 2). These results are consistent with previous inhibition tests performed in our laboratory (Rico et al. 2013; Santana-Casiano et al. 2014), where *P. tricornutum* cells were exposed to 0.31 and 0.79 µM of Cu under the same conditions described above, but with an initial cell density twice higher (2 × 10^7^ cells L^−1^). These studies showed lower growth inhibition (20 and 47.5% respectively) than those found in the present study, lower increases of polyphenol contents with respect to the control cells (only 1.3 and 2.4 times higher, respectively) and enhanced radical scavenging activities as the Cu level increased. In the current study, the highest antioxidant activities were also observed in cells exposed to increasing doses of Cu, where overproduction of MDA, commonly caused by an increase of free radicals (Danouche et al. 2022), was also detected (Table 3). Therefore, these cells must produce relevant amounts of antioxidants (such as polyphenols) in order to minimize the Cu stress by neutralizing free radicals to prevent oxidation. In fact, the MDA production is linked to the increase of phenolic compounds through a linear correlation (*r* = 0.9999; *p* < 0.05). In the same sense, Hamed et al. (2017) also observed FRAP values significantly higher in microalgae *Chlorella sorokiniana* and *Scenedesmus acuminatus* exposed to sub-lethal doses of Cu (25 and 50 µM) compared to those found in the control cells.

Accordingly, we hypothesize that the presence of high levels of Cu in the culture seawater modifies the cellular metabolism to enhance the production of polyphenols to try to minimize the toxic effect of Cu (Rocha et al. 2016; Santana-Casiano et al. 2014; Yan and Pan 2002). Among the polyphenols detected here, GAL, the most active in inhibiting DPPH (Jerez-Martel et al. 2017), experienced the maximum increase in cells exposed to Cu (up to 45.8 times greater than that found in control cells).

The increases in the production of a selected group of amino acids and polyphenols could also be the diatom cellular strategy of defense, adaptation, and tolerance to metal toxicity through their joint action. Several amino acids have shown to enhance the antioxidant effect of phenolic compounds (Ran et al. 2020; Zhang et al. 2019). In fact, more accumulation of both metabolites in barley (*Hordeum vulgare*) produced higher tolerance to the combined stress of Co and Cu (Lwalaba et al. 2020). Accumulation of amino acids and organic acids has also been reported by Jain and Chen (2018) as a defense strategy of α-proteobacterium *Caulobacter crescentus* cells stressed by Ni(II). The inhibitory effect of Ni(II) observed on the cell division rate was completely nullified by supplementation with combined amino acids and organic acids Pro, Ala, malic acid, and citric acid, with restoration of growth.

## Conclusion

This study evidenced a great accumulation of amino acids and polyphenols in *P. tricornutum* cells exposed to Cu toxicity. This accumulation was strongly influenced by the Cu concentration and could be a cellular protective mechanism for toxicity, adaptation, and tolerance. The antioxidant capacity of cells was also enhanced with increasing doses of Cu in the culture seawater, where cells also showed higher levels of MDA, indicating that these diatoms produced relevant amounts of antioxidants in response to oxidative stress, which was corroborated by the correlation between MDA and polyphenols. Pro increase observed in this study is known to confer stress tolerance by detoxifying the excess level of ROS, and Met and His accumulation may be due to their significant role in chelating and transporting of metal ions and in regulating the biosynthesis of other metabolites involved in different defense strategies. The results of the current manuscript will help to understand how the marine diatoms respond to Cu toxicity by producing amino acids and polyphenols and will support potential candidates to study and understand their role in regulating heavy metals in seawater as components of the dissolved organic matter.

## Supplementary Information

Below is the link to the electronic supplementary material.

Supplementary file1 (PDF 137 KB)

## Data Availability

Data are available from the authors upon reasonable request. The analysis of variance (ANOVA) table for concentration of amino acids in *Phaeodactylum tricornutum* cells exposed to different Cu treatments during different times is available as supplementary data.
