# Supplementary material for: Copper toxicity leads to accumulation of free amino acids and polyphenols in Phaeodactylum tricornutum diatoms
Source: Environ Sci Pollut Res Int. 2023 Feb 21;30(17):51261–70. doi: 10.1007/s11356-023-25939-0 (PMC10104907; doi:10.1007/s11356-023-25939-0)
Supplement: Supplementary file 1 — Supplementary file1 (PDF 137 KB) [file 11356_2023_25939_MOESM1_ESM.pdf]

Supplementary data

Article title: **Copper toxicity leads to accumulation of free amino acids and polyphenols in *Phaeodactylum tricornutum* diatoms**

Journal name: Environmental Science and Pollution Research

Author names: Paula Santiago-Díaz, Argimiro Rivero, Milagros Rico*, Aridane González González, Melchor González-Dávila, Magdalena Santana-Casiano

*Corresponding author at: Departamento de Química, Facultad de Ciencias del Mar, Universidad de Las Palmas de Gran Canaria, Campus de Tafira, 35017 Las Palmas de Gran Canaria, Canary Islands, Spain
Corresponding author e-mail: milagros.ricosantos@ulpgc.es

Table 4 shows significant differences between Cu treatments for each amino acid after 12 and 18 days. Tukey's test was performed to determine significant differences (considered at $p<0.05$) between each treatment and the control for individual amino acid.

Table 4. Analysis of variance (ANOVA) table for concentration of amino acids in *Phaeodactylum tricornutum* cells exposed to different Cu treatments during different times in days.

Amino acid	Days	Sum of squares	Df	Mean square	F-value	p-value
His	12	5205	3	1735	5236	< 0.05
His	18	13533	3	4511	3807	< 0.05
His	21	1922	3	641	10582	< 0.05
Arg	12	1.63	2	0.813	23	< 0.05
Arg	18	360	2	180	22737	< 0.05
Arg	21	4.38	3	1.46	162	< 0.05
Glu	12	27.4	3	9.13	11364	< 0.05
Glu	18	133	3	44.5	21188	< 0.05
Glu	21	4.70	3	1.57	893	< 0.05
Asp	12	89.4	3	29.8	57600	< 0.05
Asp	18	-	-	-	-	-
Asp	21	15.1	3	5.03	5644	< 0.05
Pro	12	12.7	3	4.23	222	< 0.05
Pro	18	11.5	3	3.83	822	< 0.05
Pro	21	3.74	3	1.25	146	< 0.05
Met	12	37461	3	12487	638	< 0.05
Met	18	5612	3	1871	426	< 0.05
Met	21	10886	3	3629	409	< 0.05
Val	12	0.509	1	0.509	139375	< 0.05
Val	18	31.0	2	15.5	1308	< 0.05
Val	21	5.77	3	1.92	900	< 0.05
Lys	12	0.272	1	0.272	274	< 0.05
Lys	18	0.114	1	0.114	233	< 0.05
Lys	21	9.66	2	4.83	6568	< 0.05
